# Flindissone, a Limonoid Isolated from *Trichilia prieuriana*, Is an LXR Agonist

**DOI:** 10.1021/acs.jnatprod.3c00059

**Published:** 2023-08-01

**Authors:** Mirta Resetar, Borris R. Tietcheu Galani, Armelle T. Tsamo, Ya Chen, Daniel Schachner, Stefanie Stolzlechner, Julio I. Mawouma Pagna, Mehdi A. Beniddir, Johannes Kirchmair, Verena M. Dirsch

**Affiliations:** † Department of Pharmaceutical Sciences, Division of Pharmacognosy, University of Vienna, Josef-Holaubek-Platz 2, 1090 Vienna, Austria; ‡ Department of Biological Sciences, Faculty of Science, University of Ngaoundere, PO Box 454, Ngaoundere, Adamawa, Cameroon; § Department of Organic Chemistry, Faculty of Science, University of Yaounde I, PO Box 812, Yaounde, Cameroon; ⊥ Department of Pharmaceutical Sciences, Division of Pharmaceutical Chemistry, University of Vienna, Josef-Holaubek-Platz 2, 1090 Vienna, Austria; ∥ Center for Cancer Research, Medical University of Vienna, Borschkegasse 8a, 1090 Vienna, Austria; ∇ Équipe “Chimie des Substances Naturelles” BioCIS, CNRS, Université Paris-Saclay, 17 Avenue des Sciences, 91400 Orsay, France

## Abstract

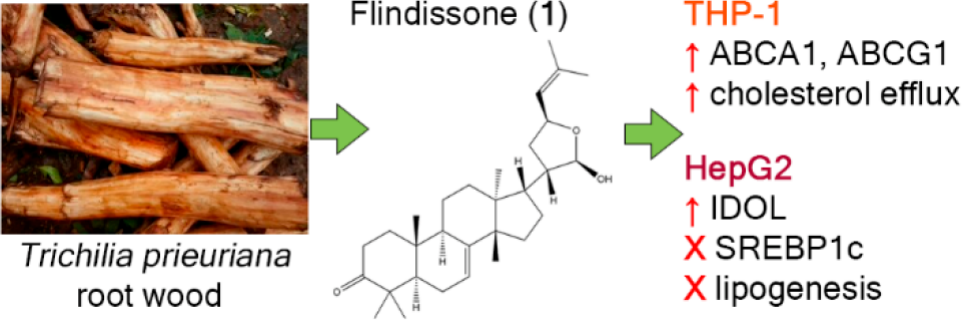

In this study, the ability of six limonoids from *Trichilia prieuriana* (Meliaceae) to activate the liver X receptor (LXR) was assessed. One of these limonoids, flindissone, was shown to activate LXR by reporter-gene assays. Flindissone is a ring-intact limonoid, structurally similar to sterol-like LXR ligands. In endogenous cellular settings, flindissone showed an activity profile that is characteristic of LXR agonists. It induced cholesterol efflux in THP-1 macrophages by increasing the cholesterol transporter ABCA1 and ABCG1 gene expression. In HepG2 cells, flindissone induced the expression of IDOL, an LXR-target gene that is associated with the downregulation of the LDL receptor. However, unlike synthetic and similarly to sterol-based LXR agonists, flindissone did not induce the expression of the SREBP1c gene, a major transcription factor regulating de novo lipogenesis. Additionally, flindissone also appeared to be able to inhibit post-translational activation of SREBP1c. The results presented here reveal a natural product as a new LXR agonist and point to an additional property of *T. prieuriana* and other plant extracts containing flindissone.

*Trichilia prieuriana* A. Juss is an evergreen tree that grows in tropical Africa. It belongs to the genus *Trichilia* within the Meliaceae family. Leaves, stem, and root bark as well as root wood preparations have reported medicinal applications in treating various conditions, particularly bacterial and parasitic infections and pain or as a purgative.^1^ Members of the Meliaceae family characteristically produce limonoids, a group of highly oxygenated and modified triterpenes, as secondary metabolites.^2^ The prototypical limonoid structure (ring-intact skeleton) consists of a tetracyclic triterpene (A/B/C/D rings) and a 17β-furan ring, although the majority of the naturally occurring limonoids possess extensively rearranged skeletons.^2^ Limonoids from the genus *Trichilia* were reported to have insecticidal, anti-inflammatory, and cytotoxic activities.^3^ Pagna et al. isolated and identified chemical constituents of *T. prieuriana*, which included the limonoids flindissone (**1**), deoxyflindissone (**2**), picraquassin E (**3**), and prieurianin (**4**). In their study, **1** showed strong antibacterial activity.^4^

The liver X receptor (LXR) is a member of the nuclear receptor family of ligand-activated transcription factors. It consists of three main domains: the N-terminal domain with a ligand-independent transactivation function, the DNA-binding domain (DBD), and the ligand-binding domain (LBD). To be transcriptionally active, the LXR has to form a heterodimer with the retinoid X receptor (RXR), which can also permissively activate the LXR heterodimer through its own ligand. There are two LXR isotypes, which differ mainly in their pattern of expression, with LXRα being the predominant form in the liver, intestine, and macrophages, while LXRβ is ubiquitously expressed.^5^ The endogenous ligands for LXR are most likely oxidized cholesterol derivatives, such as 22*R*-hydroxycholesterol (22*R*-OHC),^6,7^ given that the LXR is a major regulator of cellular and whole-body cholesterol homeostasis.^8,9^ LXR regulates cholesterol absorption in the intestine,^10^ reverse cholesterol transport from peripheral tissues and macrophages,^11,12^ and cholesterol uptake by the liver.^13,14^ Conditions such as atherosclerosis are characterized by the accumulation of cholesterol-laden macrophages inside the vessel wall. Targeting LXR is considered a viable option to increase cholesterol efflux from atherosclerotic macrophages.^15,16^ However, synthetic LXR agonists, unlike endogenous oxysterols, induce hepatic steatosis and hypertriglyceridemia, due to activation of the LXR-SREBP1c pathway for de novo lipogenesis in the liver.^10,17,18^ In addition, the liver LDL receptor (LDLR) is subjected to negative regulation by LXR.^14,19^ Systemic LXR agonism can thus potentially lead to reduced LDL uptake by the liver and an increase in the blood levels of atherogenic LDL particles.^14,18,19^

Limonoids isolated from *T. prieuriana*, especially those with a ring-intact structure (**1**–**3**, Chart 1), share a four-ring core structure similar to sterol-like LXR ligands. Among those with reported agonistic activity are desmosterol,^20,21^ a precursor in the cholesterol synthesis pathway, endogenous and synthetic oxysterols, and other sterol derivatives,^6,7,22−29^ as well as natural products of the triterpene type.^30−33^ This prompted us to investigate whether some limonoids are LXR agonists as well.

**Chart 1:**
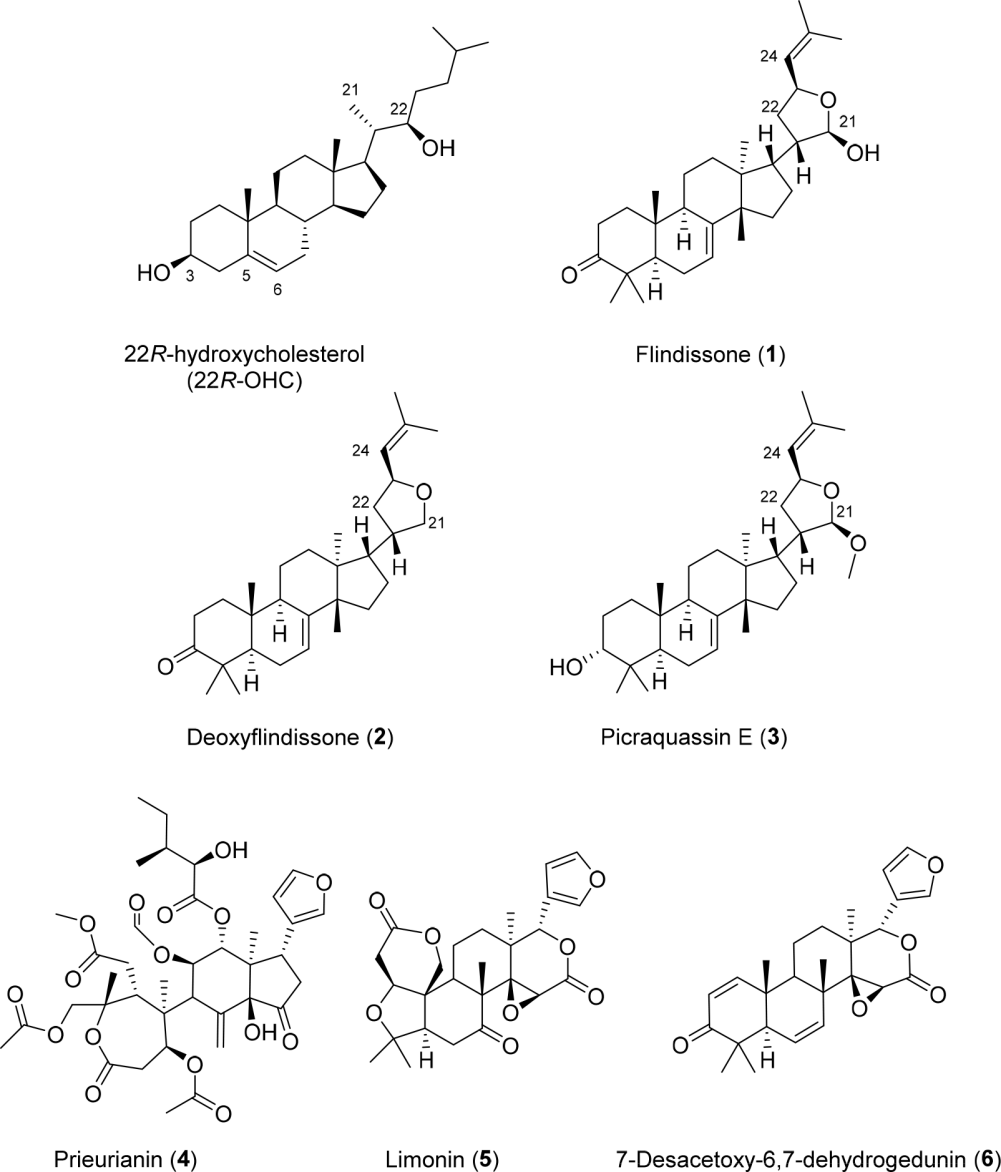
Structures of the Compounds Explored in This Study

## Results and Discussion

**Figure 1:**
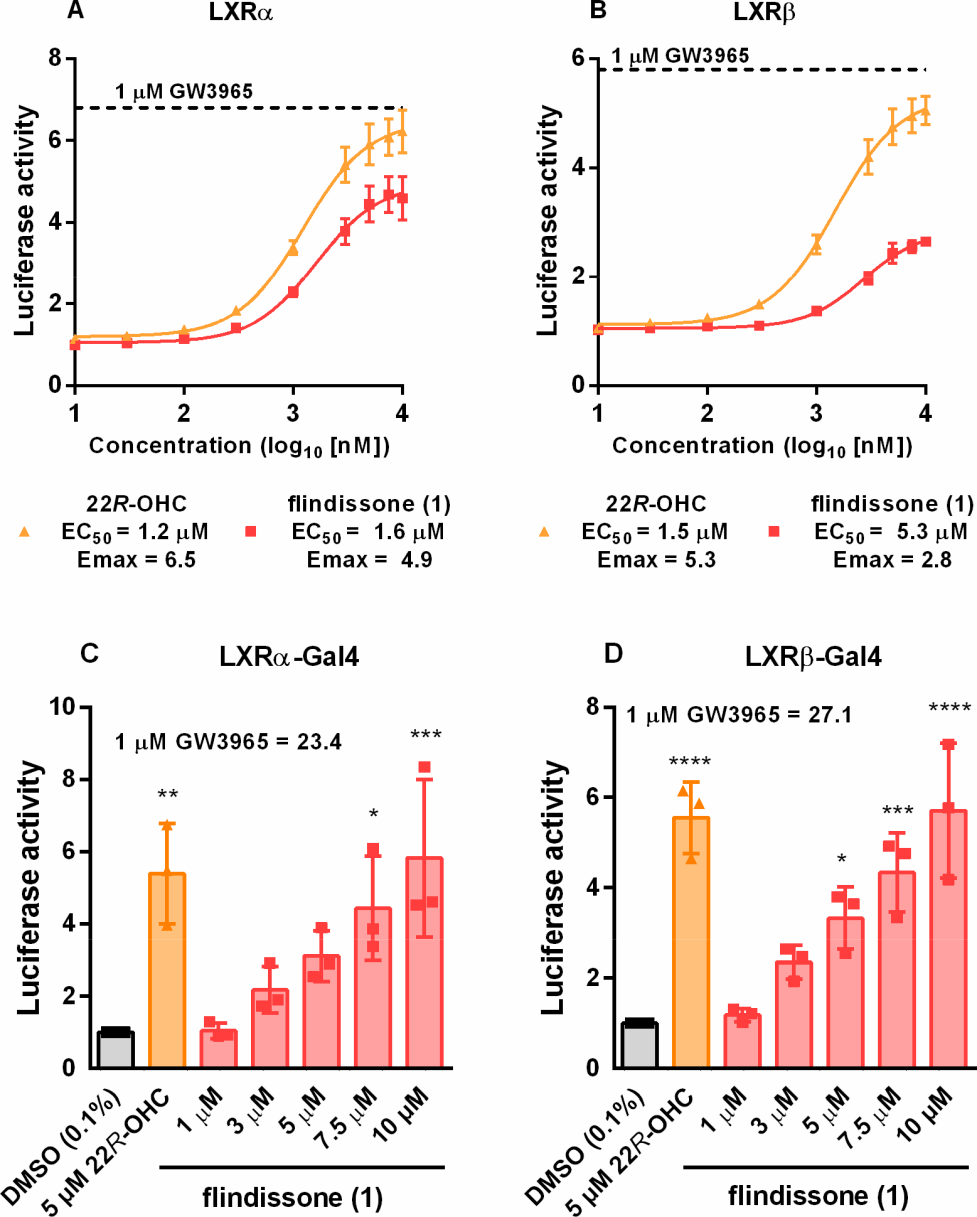
Concentration–response curves for flindissone (**1**) and 22*R*-hydroxycholesterol (22*R*-OHC) activating the LXRα (A) and LXRβ (B) receptors in luciferase assays employing full-length receptors. Data points show mean ± SEM, *N* = 4. EC_50_ and *E*_max_ values were obtained by the nonlinear fitting of the log-transformed concentrations using the variable slope. Activation of LXRα-Gal4 (C) and LXRβ-Gal4 (D). Bars show mean, error bars SD, and data points individual values. *N* = 3. One-way ANOVA with Dunnett’s post hoc test. Significance indicated in comparison to the DMSO (0.1%) control: *: *p* = 0.05, **: *p* = 0.01, ***: *p* = 0.001, ****: *p* = 0.0001, no indication = not significant.

The six limonoids **1**–**6** were assessed in luciferase assays for their ability to activate LXRα or LXRβ at concentrations between 1 and 10 μM. Only **1** was able to activate LXRs beyond the 2-fold threshold, while **3**, **4**, and **6** showed significant cytotoxicity (data not shown). Therefore, the subsequent characterization focused on **1** alone. Complete concentration–response curves of **1** in LXRα and LXRβ luciferase assays are shown in Figures 1A and 1B, respectively. In comparison to the steroid LXR agonist 22*R*-OHC, **1** showed comparable potency and a somewhat smaller efficacy in activating the LXRα receptor, but it was much less effective in activating LXRβ. To verify that **1** binds to the LXR-LBDs, Gal4-luciferase assays were employed. The Gal4-luciferase assay requires the yeast response element UAS, which cannot be activated via cell-endogenous pathways. Additionally, a permissive contribution of RXR to the activation of the LXR-response element can be excluded. Therefore, the activation of the hybrid receptor is an indication that the ligand binds directly to the LBD of the receptor. In comparison to the synthetic agonist GW3965, both steroid structures were much less effective in activating the Gal4 receptors but comparable to one another (Figures 1C and 1D). Compound **1** could activate both the LXRα- and LXRβ-Gal4 receptors in a concentration-dependent manner. However, a full sigmoidal curve could not be obtained due to the emergence of toxicity at concentrations higher than 10 μM (Figure S6, Supporting Information). Finally, **1** did not activate other nuclear receptors: RXR, FXR, or RORγ (data not shown).

**Figure 2:**
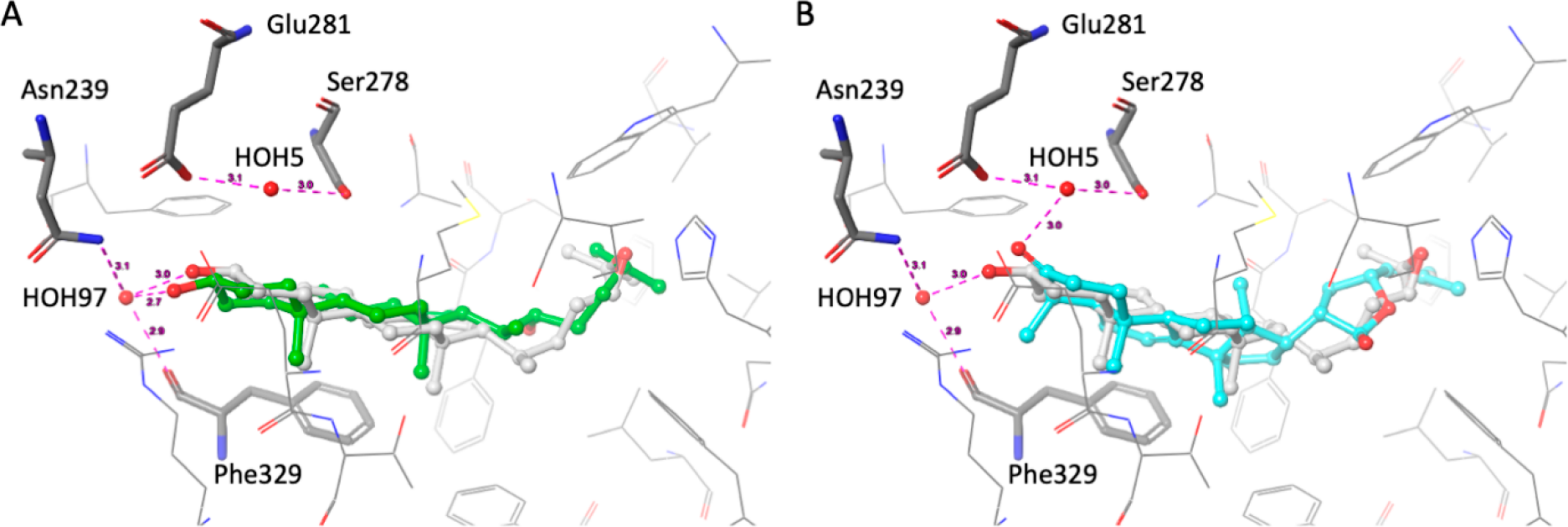
Depiction of the likely binding mode of (A) 22*R*-hydroxycholesterol (22*R*-OHC; green carbon atoms) and (B) flindissone (**1**; cyan carbon atoms) for the LXRβ ligand binding domain, derived by docking. 24(*S*),25-Epoxycholesterol (eCH), the ligand present in the cocrystal structure used for docking (PDB 1P8B),^34^ is depicted with gray carbon atoms. Magenta dashed lines indicate hydrogen bond interactions; the numbers indicate distances in Å. Amino acid residues forming hydrogen bonds with the ligands via H_2_O molecules are marked by the thick tube representation.

The X-ray crystal structure of the human LXRβ-LBD in complex with 24(*S*),25-epoxycholesterol (eCH), a potent endogenous oxysterol activator of LXR, reveals the binding mode of steroid LXR agonists to the ligand binding pocket (PDB 1P8D).^34^ Compound **1** likely binds to the LXR-LBD in a mode similar to eCH and 22*R*-OHC. The likely binding modes of **1** and 22*R*-OHC for the LXRβ-LBD were derived by in silico docking with Glide.^35−37^ Both **1** and 22*R*-OHC produced stable poses that are similar to the experimentally observed binding mode of eCH (Figure 2). The Glide docking scores (i.e., estimated binding energies) were −9.65 and −10.80 kcal/mol for **1** and 22*R*-OHC, respectively, indicating good geometric matches of the predicted poses with the ligand binding site (the Glide docking score for redocked eCH is −10.74 kcal/mol). The hydroxyl moiety in position 3 of 22*R*-OHC is predicted to form hydrogen bonds with Asn239 and Phe329 via the H_2_O molecule HOH97. This is consistent with the hydrogen bonds observed for eCH in the X-ray structure. The presence of two methyl groups in **1** at position 4 hinders the formation of hydrogen bonds. Instead, the keto group in position 3 of **1** is likely to form hydrogen bonds with Ser278 and Glu281 via the H_2_O molecule HOH5 (Figure 2). The hydroxyl group in position 21 of **1** likely also plays a role in its bioactivity since **2**, which lacks this group, was not active in luciferase assays. However, the role of this particular hydroxyl moiety in LXR binding could not be conclusively assessed with docking. It is also plausible that the hydroxyl group influences compound **1**’s biochemical properties such as intracellular transport and metabolism and, as a consequence, its availability for target binding.

**Figure 3:**
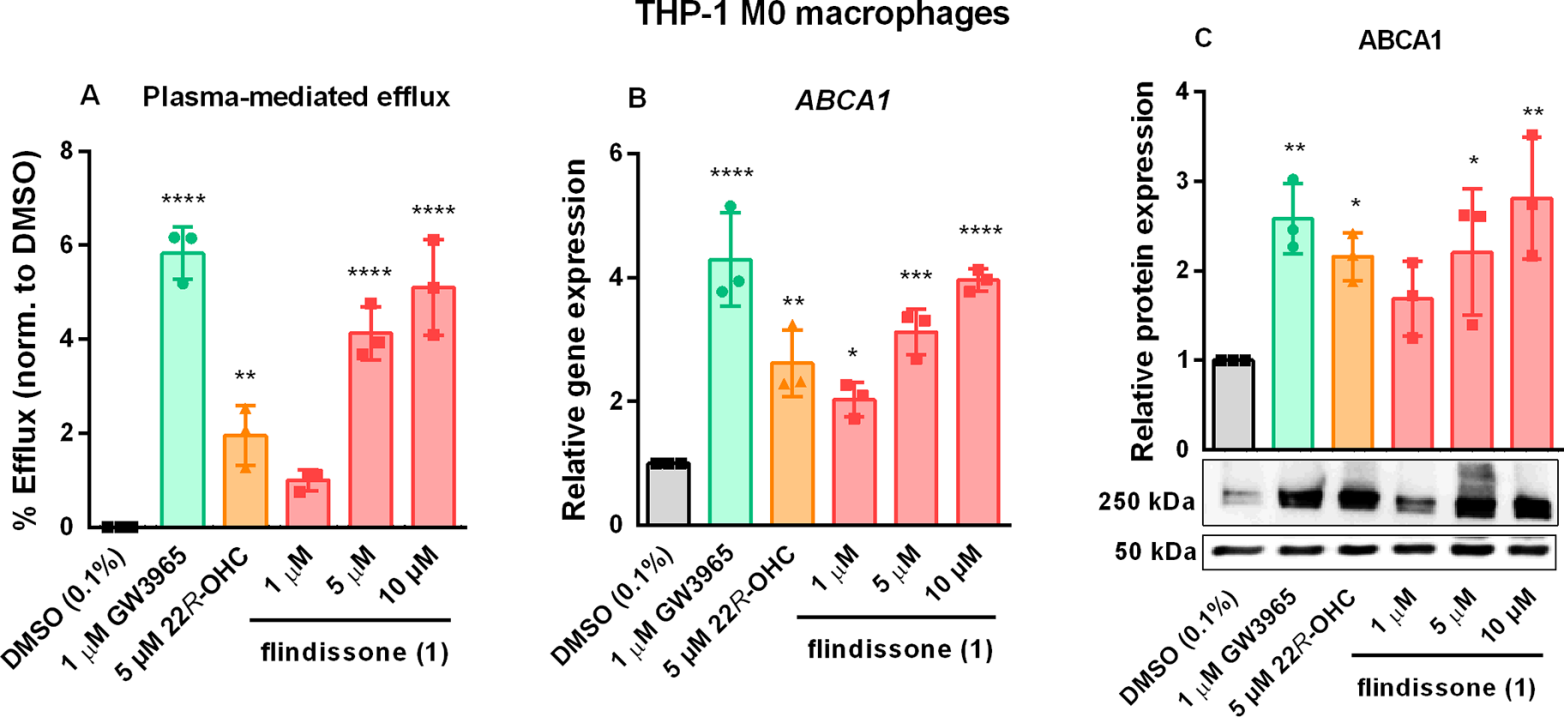
Flindissone (**1**)-mediated stimulation of cholesterol efflux in THP-1 M0 macrophages (A). The gene expression (mRNA level) of the cholesterol transporter ABCA1, measured by RT-qPCR (B) and its protein quantification with the representative blot below (C). Upper panel: 250 kDa: ABCA1. Lower panel: 50 kDa: α/β-tubulin. For all graphs: bars show mean, error bars SD, and data points individual values. *N* = 3. One-way ANOVA with Dunnett’s post hoc test. Significance indicated in comparison to the DMSO (0.1%) control: *: *p* = 0.05, **: *p* = 0.01, ***: *p* = 0.001, ****: *p* = 0.0001, no indication = not significant.

Next, the ability of **1** to induce the gene expression of known LXR targets in an endogenous cellular environment was examined. LXR agonists are known regulators of the macrophage cholesterol efflux, where they upregulate gene expression of the cholesterol transporters ABCA1 and ABCG1.^11,12^ In the THP-1 macrophage cell line, **1** was able to stimulate plasma-mediated cholesterol efflux in a concentration-dependent manner (Figure 3A). At 5 μM concentration, **1** was more effective than 22*R*-OHC. **1** induced transcription of the ABCA1 gene (Figure 3B), which was accompanied by the corresponding increase in ABCA1 protein levels (Figure 3C). In addition, the ABCG1 gene was upregulated by **1** (Figure S7, Supporting Information).

**Figure 4:**
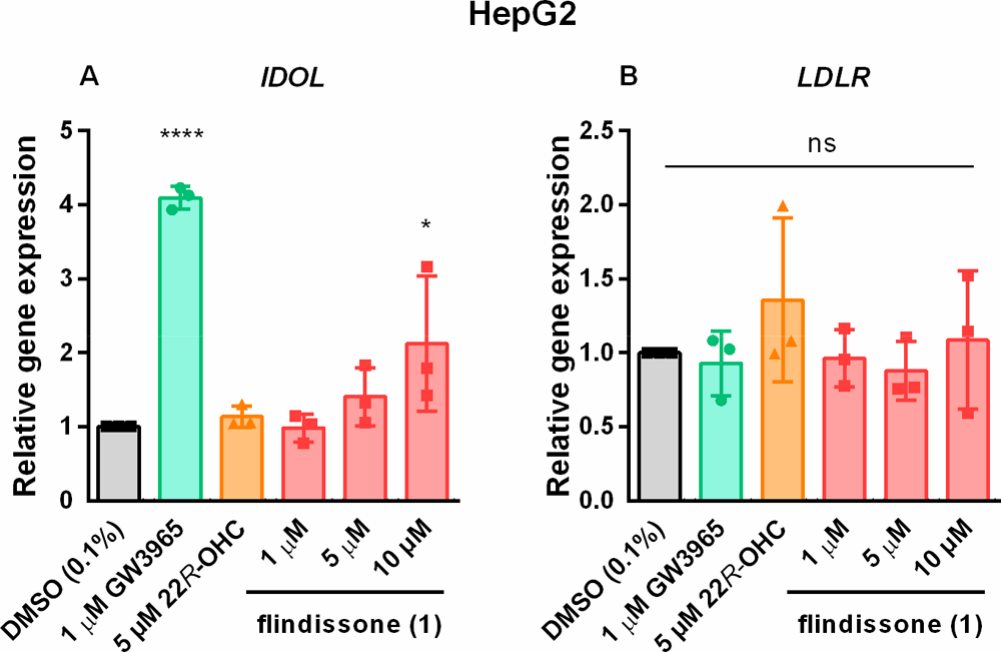
Flindissone-mediated target-gene regulation in the HepG2 cell line. The mRNA levels of IDOL (A) and LDLR (B) were measured by RT-qPCR. *N* = 3. Bars show mean ± SD. One-way ANOVA with Dunnett’s post hoc test. Significance indicated in comparison to the DMSO (0.1%) control: *: *p* = 0.05, ****: *p* = 0.0001, ns = not significant.

In the liver, LXR activation regulates cholesterol uptake. LXR agonists have been shown to decrease protein levels of the LDL receptor and thus reduce LDL uptake.^14,19^ The mechanism behind it involves transcriptional upregulation of the inducible degrader of the LDLR (IDOL; official name MYLIP) gene, which is a direct LXR target.^14^ In the hepatoma cell line HepG2, **1** was not as potent or effective as GW3965, with only the highest concentration of 10 μM inducing a significant increase in IDOL expression (Figure 4A). None of the LXR agonists had any effect on the LDLR mRNA levels (Figure 4B). Unfortunately, it was not possible to detect the LDLR protein using Western blot in the HepG2 cell line due to the inconsistency of the employed antibodies (data not shown) and thus determine the effect of **1** on the LDLR protein levels. However, based on the published literature,^14,19^ the LDLR protein levels are supposed to be decreased with GW3965 and 10 μM **1** treatments.

Increased lipogenesis in the liver is a major side-effect of LXR activation.^10,17,18^ LXR agonists increase the expression of SREBP1c (SREBF1 gene), which is the master transcriptional regulator of fatty acid synthesis. For an active state, SREBP1c has to be cleaved from its 130 kDa precursor form located in the ER membrane to an active 60 kDa protein that can translocate into the nucleus.^38^ Unlike synthetic LXR agonists, sterol-like LXR ligands appear to be weaker activators of the SREBP1c transcription, particularly in liver cells,^24,26,27,29,32^ although some ligands can also selectively upregulate SREBP1c expression^39^ or function as context-specific antagonists.^40^ Furthermore, sterols and oxysterols were shown to inhibit the proteolytic activation of SREBP1c by binding to the SREBP cleavage-activating protein (SCAP) and insulin-induced gene 2 protein (INSIG2), respectively. In a sterol-bound state, these two chaperones anchor the inactive SREBP1c precursor to the ER membrane.^41,42^

**Figure 5:**
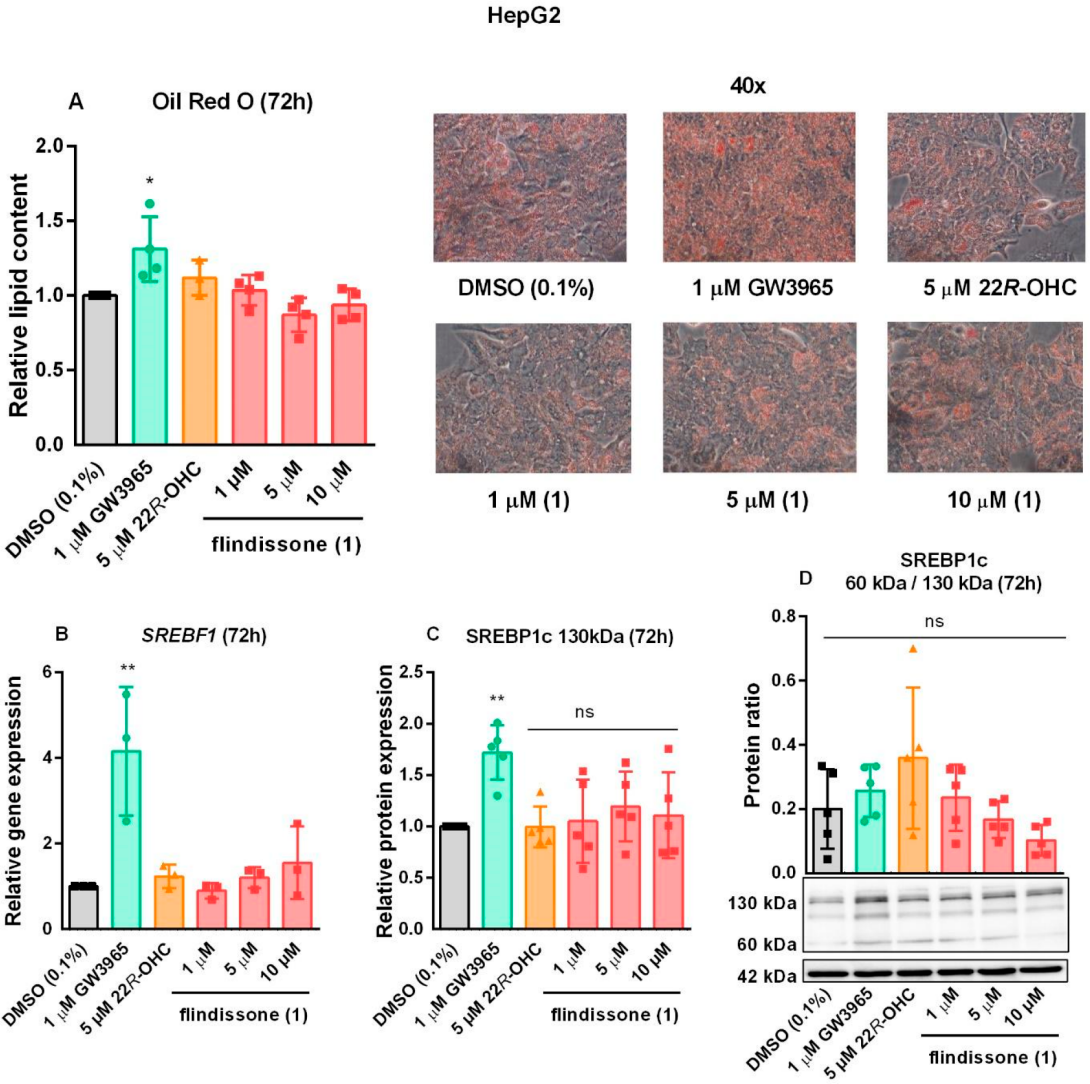
Flindissone-mediated regulation of lipogenesis in HepG2 cells. Measurements of the cellular neutral lipid content by Oil Red O staining (A). *N* = 4. Representative images were taken at 40× magnification. Gene expression (mRNA) of SREBF1 (B), measured by RT-qPCR. *N* = 3. Quantification of the SREBP1c 130 kDa (C) and cleaved 60 kDa product relative to its 130 kDa precursor (D). Representative blots of the SREBP1c protein levels below. Upper panel: 130 kDa: SREBP1c precursor; 60 kDa: cleaved SREBP1c. Lower panel: 42 kDa: actin. For all graphs: Bars show mean, error bars SD, and data points individual values. One-way ANOVA with Dunnett’s post hoc test. Significance indicated in comparison to the DMSO (0.1%) control: *: *p* = 0.05, **, *p* = 0.01, no indication or ns = not significant.

In HepG2 cells, only the synthetic agonist GW3965 induced an increase in cellular lipid content, as measured by the Oil Red O staining (Figure 5A). In comparison to GW3965, **1** significantly induced neither SREBF1 transcription (Figure 5B) nor increased protein levels of the 130 kDa precursor at any concentration (Figure 5C). However, **1** appeared to inhibit proteolytic processing of the SREBP1c, as shown by the concentration-dependent reduction of the 60 to 130 kDa protein ratio after 72 h of compound treatment, although the effects were not statistically significant (Figure 5D). In addition to SREBP1c, a similar proteolytic-activation mechanism also applies to SREBP2, a transcription factor involved in cholesterol synthesis.^41,42^ Since it was not possible to detect cleaved SREBP2 protein in HepG2 cells sufficiently to allow for quantification (data not shown), expression of its target gene 3-hydroxy-3-methylglutaryl-coenzyme A reductase (HMGCR) was measured instead. Compound **1** dose-dependently also decreased HMGCR expression (Figure S8, Supporting Information). 22*R*-OHC was reported to inhibit proteolytic processing of the SREBP2 protein at 20 μM;^42^ however no inhibition of the proteolytic processing of SREBP1c nor a decrease in HMGCR gene expression could be observed here up to concentrations of 10 μM (data not shown). Since the nonsteroidal agonist GW3965 also reduced the HMCGR gene expression, the mechanism involved may not depend solely on the inhibition of SREBP2 proteolytic processing.

Taken together, these observations demonstrate that flindissone (**1**) is a new natural product, an LXR agonist. In vitro characterization of **1** showed an activity profile that corroborates its function as an LXR agonist. Like GW3965 and 22*R*-OHC, **1** induced ABCA1 and ABCG1 gene expression and cholesterol efflux from THP-1 M0 macrophages. In HepG2 cells, GW3965 and **1** induced IDOL expression, which is associated with the downregulation of LDL uptake in the liver. However, unlike GW3965, but similarly to 22*R*-OHC, **1** did not significantly induce SREBF1 expression and subsequent activation of de novo lipogenesis. In addition, **1** appears to be able to inhibit the proteolytic processing of SREBP transcription factors, most likely due to its structural similarity to sterols. However, **1** was not particularly potent in cellular assays, as the effects were most notable above 1 μM. This may be due to its hydrophobic nature, as such compounds form micelles, which reduces their availability for target binding. Nonetheless, oral administration of sterol-like LXR ligands in animal studies was sufficient to detect them in various tissues and observe their LXR-mediated effects.^23,24,43^ Even in the absence of systemic effects, orally administered sterol-based LXR ligands were shown to be active in enterocytes,^23,24^ where LXR activation reduces cholesterol absorption and induces cholesterol efflux.^9,10^ The results presented here uncover a potential antiatherogenic benefit of *T. prieuriana* extracts, as well as other plants containing significant amounts of **1**, but also point to possible disadvantages of such extracts originating from activation of LXR in the liver.

## Experimental Section

### General Experimental Procedures

IR spectra were recorded on a Bruker Fourier transform/infrared (ATR) spectrophotometer. Exact mass HRESIMS data were recorded using an Agilent 6546 QTOF mass spectrometer (Agilent Technologies, Massy, France) equipped with an ESI source, operating in both positive- and negative-ion mode. A BEH Acquity C18 UPLC column (2.1 × 100 mm; i.d. 1.8 μm, Waters) was used, with a flow rate of 0.5 mL·min^–1^ and a linear gradient from 5% B (A: H_2_O + 0.1% formic acid, B: MeCN + 0.1% formic acid) to 100% B over 15 min. Source parameters were set as follows: capillary temperature at 320 °C, source voltage at 3500 V, and sheath gas flow rate at 11 L·min^–1^. The divert valve was set to waste for the first 3 min. MS scans were operated in full-scan mode from *m*/*z* 100 to 1200 (0.1 s scan time) with a mass resolution of 67,000 at *m*/*z* 922. In the positive-ion mode, purine (C_5_H_4_N_4_) [M + H]^+^ ion (*m*/*z* 121.050873) and the hexakis(1*H*,1*H*,3*H*-tetrafluoropropoxy)phosphazene (C_18_H_18_F_24_N_3_O_6_P_3_) [M + H]^+^ ion (*m*/*z* 922.009798) were used as internal lock masses. LC-DAD-ELSD analysis of flindissone (**1**) was conducted on an HPLC (Agilent) equipped with an ELSD (Agilent) using an XSELECT column (4.6 mm × 150 mm, i.d., 5 μm, Waters) with a flow rate of 1 mL·min^–1^ and a linear gradient from 30% B (A: H_2_O + 0.1% formic acid, B: MeCN + 0.1% formic acid) to 100% B over 15 min. 1D NMR spectra were recorded in deuterated chloroform on an AVANCE 300 MHz NMR spectrometer (proton ^1^H at 300 MHz and carbon ^13^C at 75 MHz). Chemical shifts δ are referenced to residual solvent signals and reported in parts per million (ppm) relative to tetramethylsilane (TMS), and coupling constants *J* are given in Hz. Column chromatography (CC) was performed using Merck MN silica gel 60 Mesh (0.04–0.063 nm), and thin layer chromatography (TLC) was performed on aluminum silica gel 60 F254 (Merck) precoated plates (0.2 mm layer thickness). Spots were visualized on TLC by a UV lamp (254 and 365 nm) or by heating after spraying with a 20% H_2_SO_4_ (v/v) solution. Different mixtures of *n*-hexane, EtOAc, and MeOH were used as eluting solvents.

### Plant Material

The root wood of *T. prieuriana* was collected in August 2016 by Dr. Nole (plant taxonomist at the Institute of Medical Research and Medicinal Plants Studies) in Mindourou, East Region, Cameroon. Latitude: 3.98250 N; longitude: 14.89501 E (approximate), elevation: 500 ft. Shortly after harvesting, the root wood was cut and air-dried at 28 °C for 4 weeks. The plant material was identified by comparison with the specimen available at the National Herbarium of Cameroon under voucher number 66990/HNC.

### Extraction, Fractionation, and Isolation

Dried, ground root wood (1.9 kg) of *T. prieuriana* was extracted three times with EtOH–H_2_O (7:3) at room temperature (28 °C) for 48 h. The resulting solutions were filtered, combined, and evaporated to dryness under reduced pressure at 40 °C, yielding 387 g of brown crude extract. A 145 g amount of the hydro-ethanolic crude extract obtained from the root wood was subjected to flash chromatography over silica gel using a mixture of *n*-hexane and AcOEt of increasing polarity to afford four main fractions, labeled F1 (20 g; *n*-hexane–AcOEt, 4:1), F2 (10 g; *n*-hexane–AcOEt, 1:1), F3 (15 g; pure AcOEt), and F4 (25 g; MeOH). Fraction F1 (20 g) was further chromatographed on a silica gel column and eluted with a mixture of *n*-hexane–AcOEt of increasing polarity. A total of 200 fractions, each of 175 mL, were collected, evaporated, and combined based on their TLC profiles to afford seven main subfractions [S1 (1–30), S2 (31–60), S3 (61–86), S4 (87–115), S5 (116–148), S6 (149–180), and S7 (181–200)]. Series S1, S2, and S3, after precipitation at room temperature, followed by filtration and washing with a mixture of *n*-hexane–AcOEt (1:1), yielded compounds prieurianin (**4**, 20.09 mg), flindissone (**1**, 2 mg), and picraquassin E (**3**, 10.68 mg) respectively. Fraction F2 (10 g), obtained by elution with *n*-hexane–AcOEt (1:1), was further purified over a silica gel column using a gradient of *n*-hexane–AcOEt to give a total of 160 fractions of 100 mL each, which were further combined based on TLC profiles to afford nine main series [S1′ (1–15), S2′ (16–35), S3′ (35–48), S4′ (49–60), S5′ (60–80), S6′ (80–105), S7′ (106–120), S8′ (106–137), and S9′ (138–160)]. Series S4′ and S5′ crystallized and, after filtration, yielded the compound deoxyflindissone (**2**, 8.56 mg).

Four additional compounds were isolated from the hydroethanolic extracts from the root wood of *T. prieuriana* by the usual chromatographic techniques. Their structures were established using a combination of 1D and 2D NMR techniques in conjunction with HRESIMS analyses and by comparison with data reported in the literature. Isolated compounds were identified as flindissone (**1**), deoxyflindissone (**2**), picraquassin E (**3**), and prieurianin (**4**).^4^ Moreover, flindissone (**1**) purity was determined at 95.554% after the integration of its LC-ELSD chromatogram (Figure S3, Supporting Information).

### Other Compounds

Limonin (**5**) was purchased from KAN Phytochemicals (Sonipat, Haryana, India), 7-desacetoxy-6,7-dehydrogedunin (**6**) and GW3965 were from Sigma-Aldrich (SML0806 and G6295, respectively), and 22*R*-OHC was from Cayman Chemical (Cay89355). All compounds were dissolved in 100% DMSO and stored at −20 °C.

### Cell Culture

HEK293T, HepG2, and THP-1 cell lines were purchased from ATCC (CRL-1573, HB-8065, and TIB-202, respectively). HEK293T and HepG2 cells were grown in DMEM with 4.5 g/L glucose (Lonza, 12-917F) and THP-1 monocytes in RPMI 1640 (Lonza, 12-167F), both supplemented with 10% fetal bovine serum (FBS) (Gibco, 26140079), 2 mM glutamine (Lonza, 17-605E), and 100 U/mL/100 μg/mL penicillin–streptomycin (Lonza, 17-602F). THP-1 monocytes were differentiated into THP-1 M0 macrophages by the addition of 200 nM PMA (Sigma-Aldrich, P8139) for 72 h.

### Luciferase Assays

Two types of luciferase assays were performed. One using the full-length human LXR receptors and a luciferase reporter gene driven by an LXR-response element from the ABCA1 gene promoter and a second assay employing hybrid receptors consisting of the LXR-LBD (ligand binding domain) and the yeast transcription factor Gal4-DBD (DNA-binding domain) where the UAS sequence is used as a promoter for luciferase expression. The plasmids used are listed in the Supporting Information. HEK293T cells were transfected with the calcium phosphate method for 6 h. For full-length LXR transfections, 3 μg of receptor, 6 μg of response element, and 3 μg of EGFP were used per 6 million cells, and for Gal4-transfections, 5 μg of Gal4-receptor, 1 μg of response element, and 3 μg of EGFP plasmid were used per 6 million cells. Transfected cells were resuspended in 2.5% charcoal-stripped FBS–DMEM, replated into a 96-well plate to a density of 50,000 cells/well, and treated with compound solutions prepared in the same medium. After an 18 h incubation period, cells were lysed with a commercial lysis buffer from Promega (E3971). Luminescence and fluorescence values were measured on a TECAN Spark Instrument (TECAN Austria, Salzburg, Austria). Luminescence was normalized by fluorescence through division and expressed relative to the vehicle (0.1% DMSO) control (“luciferase activity”). EC_50_ and *E*_max_ values were obtained by the nonlinear fitting of the log-transformed concentrations using the variable slope.

### Docking

An X-ray crystal structure of the LXRβ-LBD in complex with eCH (PDB 1P8D, resolution 2.80 Å)^34^ was utilized for docking with Glide (software version 2021-1, Schrödinger Inc., New York, NY, USA).^36^ The protein structure was prepared with the Protein Preparation Wizard within the Maestro molecular modeling environment (software version 2021-1, Schrödinger Inc.)^37^ using default settings. The preparation included the (i) addition of hydrogen atoms, (ii) assignment of bond orders, (iii) assignment of protonation and metal charge states with Epik, (iv) removal of all chains except for chain A, (v) sampling H_2_O orientations and optimization of the hydrogen bond network, and (vi) restrained minimization using the OPLS4 force field to converge heavy atoms to an RMSD of 0.30 Å. The 3D structures of flindissone and 22*R*-OHC were prepared with LigPrep within Maestro using the default settings. For docking with Glide, the ligand-binding site was defined within the Receptor Grid Generation wizard to dock ligands similar in size to the cocrystallized ligand. Glide Standard Precision (Glide SP) was used for ligand docking and up to 100 docking poses were set for output.

### Cholesterol Efflux Assay

THP-1 monocytes were plated in a 24-well plate at a density of 200.000 cells/mL per well and differentiated with 200 nM PMA for 72 h into THP-1 M0 macrophages. Macrophages were first treated with 500 μL of cholesterol solution containing 10 μg of H_2_O-soluble cholesterol (Sigma-Aldrich, C4951) and 0.1 μCi [1,2-^3^H(N)]-labeled cholesterol (PerkinElmer, NET139001MC) in 2.5% FBS DMEM medium for 24 h. Cells were washed, and 500 μL of compound solutions were added for another 24 h. Compound solutions were removed, and efflux was stimulated by the addition of 250 μL of 1% plasma in 0.1% BSA-DMEM medium. For each compound treatment, nonstimulated efflux was measured from wells where only 0.1% BSA-DMEM medium was added. After 6 h, the medium was collected and cleared by centrifugation and cells were lysed with 250 μL of 0.1N NaOH for 10 min. Media and lysates were mixed with 3.5 mL of scintillation counting liquid (PerkinElmer, 6013329). The efflux values were calculated as follows:% Efflux (1% plasma or blank) = [^3^H] (medium)/[^3^H] (medium) + [^3^H] (cells) * 100. Plasma-mediated% Efflux = % Efflux (1% plasma) – % Efflux (blank). Plasma-mediated% Efflux for compound treatments was normalized to the DMSO by subtraction.

### Compound Treatments

For RNA and protein extractions, 800.000 THP-1 macrophage cells plated in six-well plates (after 72 h differentiation) were first treated with 2 mL of cholesterol solution containing 40 μg of H_2_O-soluble cholesterol in 2.5% FBS–DMEM for 24 h before treatment with compounds, to keep consistency with the Cholesterol efflux assay. For the HepG2 cell line, 600.000 cells were plated in 4 mL per well and incubated with compounds from the following day in a 2 mL solution for 72 h, with fresh compound solution added every 24 h. All compounds were diluted in 0.1% BSA-DMEM medium to an indicated final concentration. The final concentration of the solvent (DMSO) was kept constant at 0.1% for all compounds and concentrations.

### mRNA Quantification

RNA was extracted with the innuPREP RNA Mini Kit 2.0 kit (IST Innuscreen, 845-KS-2040250) according to the manufactureŕs protocol. One μg of RNA was used for reverse-transcriptase reaction (Applied Biosystems, 4368814) and the cDNA template was diluted to a final volume of 50 μL. Two μL of the template was used for the qPCR reaction according to the manufactureŕs protocol (Promega, A6002). All primers except for the human GAPDH (Qiagen, 249900) were designed with PrimerBLAST and were tested for efficiency and specificity before use. The sequences can be found in the Supporting Information. The “relative gene expression” was calculated using the ddCt method and expressed in comparison to the 0.1% DMSO control.

### Western Blot

Cells were lysed with RIPA buffer supplemented with protease and phosphatase inhibitors, and protein was quantified using the Bradford reagent (Carl Roth, K015.1). For the detection of ABCA1 and SREBP1c, 40 and 30 μg of protein was used per lane, respectively. The following antibodies were used: ABCA1 (Novus Biologicals, NB400-105), SREBP1c (Thermo Fisher, MA5-11685), α/β-tubulin (CST, 2148), actin (mpbio, 0869100-CF) and antirabbit IgG, HRP-linked (CST, 7074). The chemiluminescence reaction was detected by a FujiFilm LAS Imager (FujiFilm, Japan) or Amersham ImageQuant 800 Fluor (Cytiva Europe, Vienna, Austria). Densitometric analyses were performed using MultiGauge (FujiFilm, Japan) or ImageQuant TL 10 (Cytiva Europe, Vienna, Austria) software. The “Relative protein expression” was obtained by normalizing the densitometric value with the one of the housekeeping protein and expressed relative to the 0.1% DMSO control.

### Oil Red O Staining

HepG2 cells were plated and treated with compounds as described in Compound treatments. Cells were fixed with 10% formaldehyde for 10 min at room temperature, rinsed with PBS, permeated with 60% isopropanol, and stained with 60% Oil Red O solution for 1 min at 37 °C. After 5 washes with PBS and drying, cells were photographed, and the dye was eluted with 100% isopropanol. OD values were measured at 492 nm, and the results were expressed relative to the DMSO sample.

### Statistical Analysis

All of the experiments were performed independently at least three times. Luciferase experiments were further performed with 4 technical replicates per experiment. Data analysis was performed in MS Excel, and graphs and statistical analysis were performed in GraphPad Prism v8 (GraphPad, La Jolla, CA, USA). For all graphs: Bars and whiskers display mean ± SD; unless otherwise indicated in the legend. Bullet points are individual values (N). Statistical test: One-way ANOVA with Dunnett’s post hoc test. Significance indicated was in comparison to the DMSO (0.1%) control.

## Supporting Information Available

Spectroscopic data of flindissone (**1**) (LC-MS (ESI+) chromatogram, HRESIMS (ESI+) spectrum, observed adducts), purity assessment of flindissone (**1**) (integration data of LC-ELSD chromatogram, ^1^H NMR and ^13^C NMR spectra); lists of plasmids used for luciferase assays and of primers used for RT-qPCR; supplementary figures (viability, ABCG1 and HMGCR gene expression) (PDF)
