# Supplementary material for: Flindissone, a Limonoid Isolated from Trichilia prieuriana, Is an LXR Agonist
Source: J Nat Prod. 2023 Aug 1;86(8):1901–9. doi: 10.1021/acs.jnatprod.3c00059 (PMC10463221; doi:10.1021/acs.jnatprod.3c00059)
Supplement: Supplementary file 1 — np3c00059_si_001.pdf [file np3c00059_si_001.pdf]

Flindissone, a Limonoid Isolated from *Trichilia prieuriana*, is an LXR Agonist

Mirta Resetar,[*,†] Borris R. Tietcheu Galani,[‡] Armelle T. Tsamo,[§] Ya Chen,[⊥] Daniel Schachner,[†] Stefanie Stolzlechner,[∥] Julio I. Mawouma Pagna,[§] Mehdi A. Beniddir,[▽] Johannes Kirchmair[⊥] and Verena M. Dirsch[†]

[†] Department of Pharmaceutical Sciences, Division of Pharmacognosy, University of Vienna, Josef-Holaubek-Platz 2, 1090 Vienna, Austria

[‡] Department of Biological Sciences, Faculty of Science, University of Ngaoundere, PO Box 454, Ngaoundere, Adamawa, Cameroon

[§] Department of Organic Chemistry, Faculty of Science, University of Yaounde I, PO Box 812, Yaounde, Cameroon

[⊥] Department of Pharmaceutical Sciences, Division of Pharmaceutical Chemistry, University of Vienna, Josef-Holaubek-Platz 2, 1090 Vienna, Austria

[∥] Center for Cancer Research, Medical University of Vienna, Borschkegasse 8a, 1090 Vienna, Austria

[▽] Équipe "Chimie des Substances Naturelles" BioCIS, CNRS, Université Paris-Saclay, 17 Avenue des Sciences - 91400 Orsay, France

Spectroscopic Data of Flindissone (1)

White powder; mp: 128-130 °C; ^{1}H NMR (300 MHz, $CDCl_3$): 1.50 ; 2.01 (2H-1 ; m); 2.71 (1H-2 ; ddd ; 14 ; 9 ; 5.5 Hz); 2.26 (1H-2´ ; m); 1.65 (1H-5 ; m); 2.31 ; 2.13 (2H-6 ; m); 5.38 (1H-7 ; m); 2.32 (H-9 ; m); 1.60 ;1.77 (2H-11 ; m); 1.58 ; 1.62 (2H-12 ; m); 1.68 (2H-15 ; m); 1.89 ; 1.38 (2H-16 ; m); 1.75 (H-17 ; m); 0.91 (3H-18 ; s); 1.01 (3H-19 ; s); 2.20 (H-20 ; m); 5.20 (1H-21 ; d ; 7 ;9 Hz); 1.75 (H-22 ; m); 1.68 (2H-22 ; m);4.78 (1H-23 ; m); 5.24 (1H-24 ; m); 1.69 (3H-26; brs); 1.70 (3H-27; brs); 0.89 (3H-28; s), 0.90 (3H-29 ; s) and 0.92 (3H-30 ; s). HRESIMS *m/z*: 419.3316 $[M+H-2H_2O]^+$ (calcd for $C_{30}H_{43}O$, 419.3313), 437.3420 $[M+H-H_2O]^+$ (base peak; calcd for $C_{30}H_{45}O_2$, 437.3419), *m/z* 477.3341 $[M+Na]^+$ (calcd for $C_{30}H_{46}O_3Na$, 477.3344).

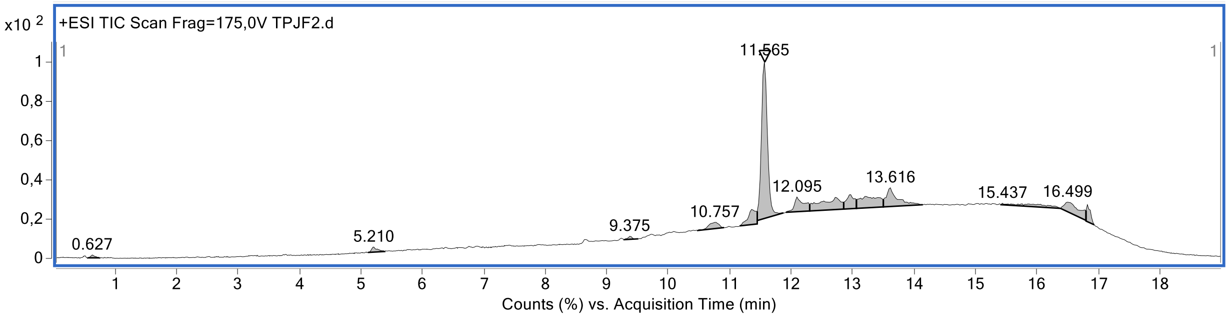

Figure S1: Representative LC-MS (ESI +) chromatogram of flindissone (**1**).

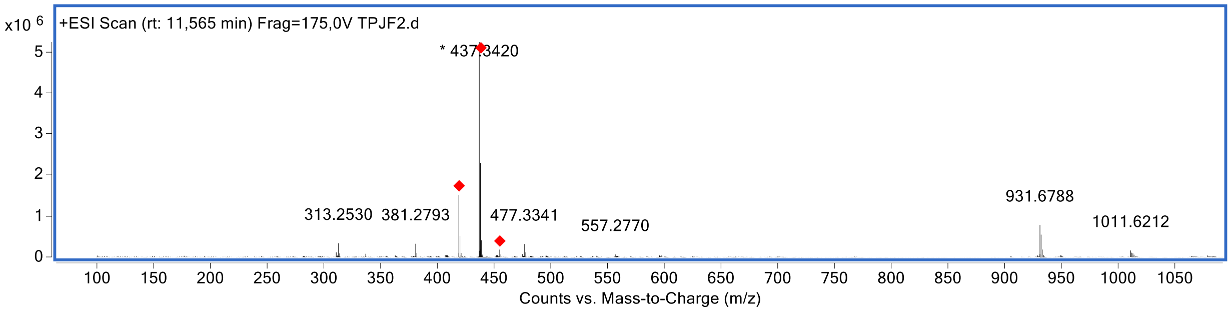

Figure S2: HRESIMS (ESI +) spectrum of flindissone (**1**).

Table S1. Observed Adducts of Flindissone (1)

Adducts	Formula	*m/z observed*	*m/z* calculated
$[M+H-2H_2O]^+$	$C_{30}H_{43}O$	419.3316	419.3313
$[M+H-H_2O]^+$	$C_{30}H_{45}O_2$	437.3420	437.3419
$[M+ Na]^+$	$C_{30}H_{46}O_3Na$	477.3341	477.3344

Purity Assessment of Flindissone (1)

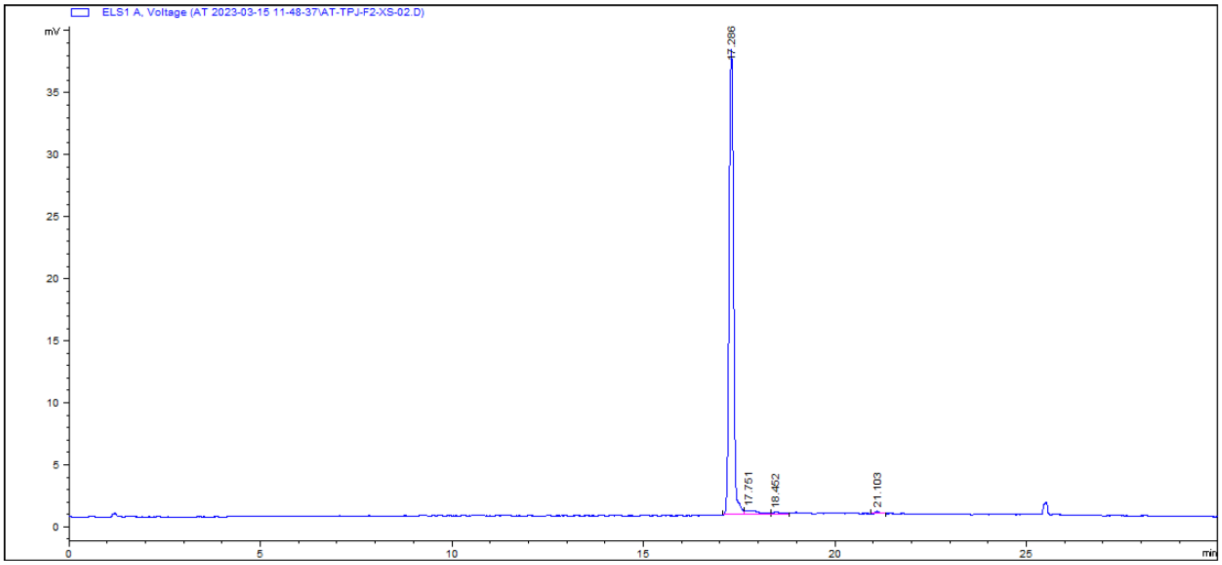

#	Time	Area	Height	Width	Area%	Symmetry
1	17.286	303.7	37.5	0.1304	95.554	0.938
2	17.751	9.3	3.5E-1	0.322	2.926	0.327
3	18.452	3.3	1.9E-1	0.2359	1.028	0.405
4	21.103	1.6	1.9E-1	0.1307	0.491	0.793

Figure S3: Integration data of LC-ELSD chromatogram of flindissone (**1**).

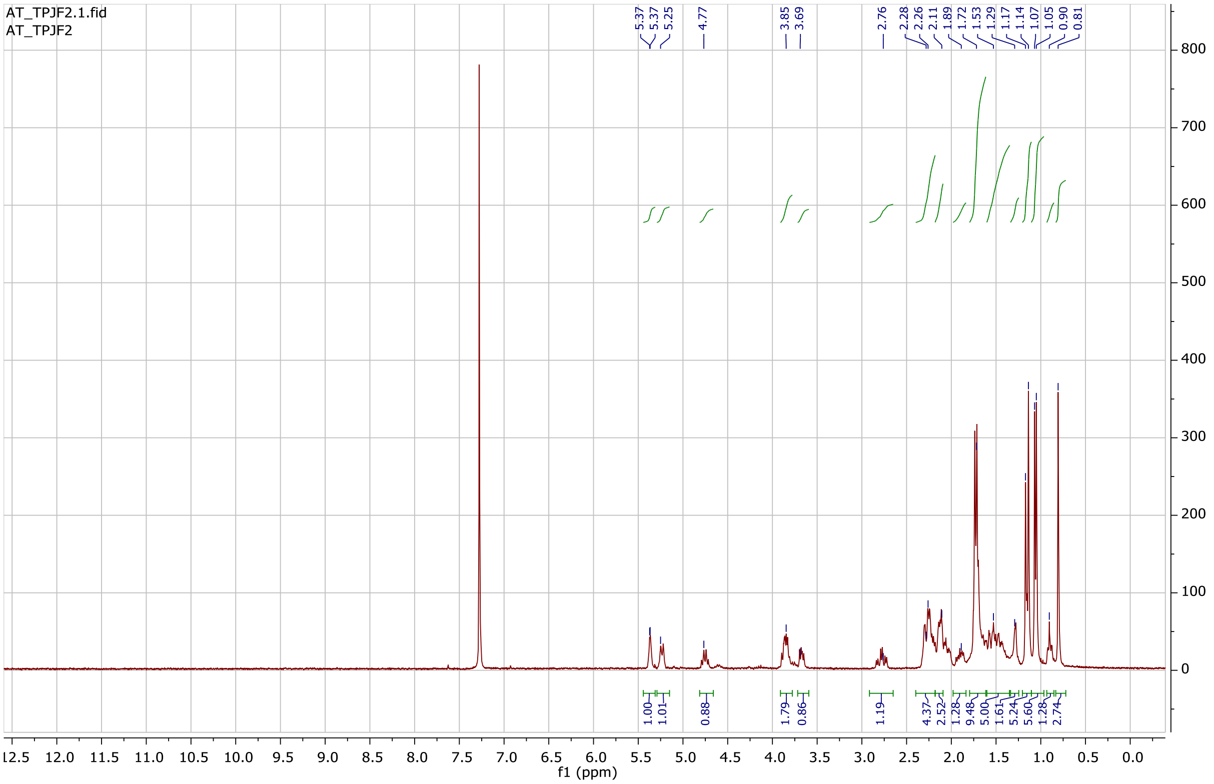

Figure S4: ^{1}H NMR spectrum of flindissone (**1**) ($CDCl_3$, 300 MHz).

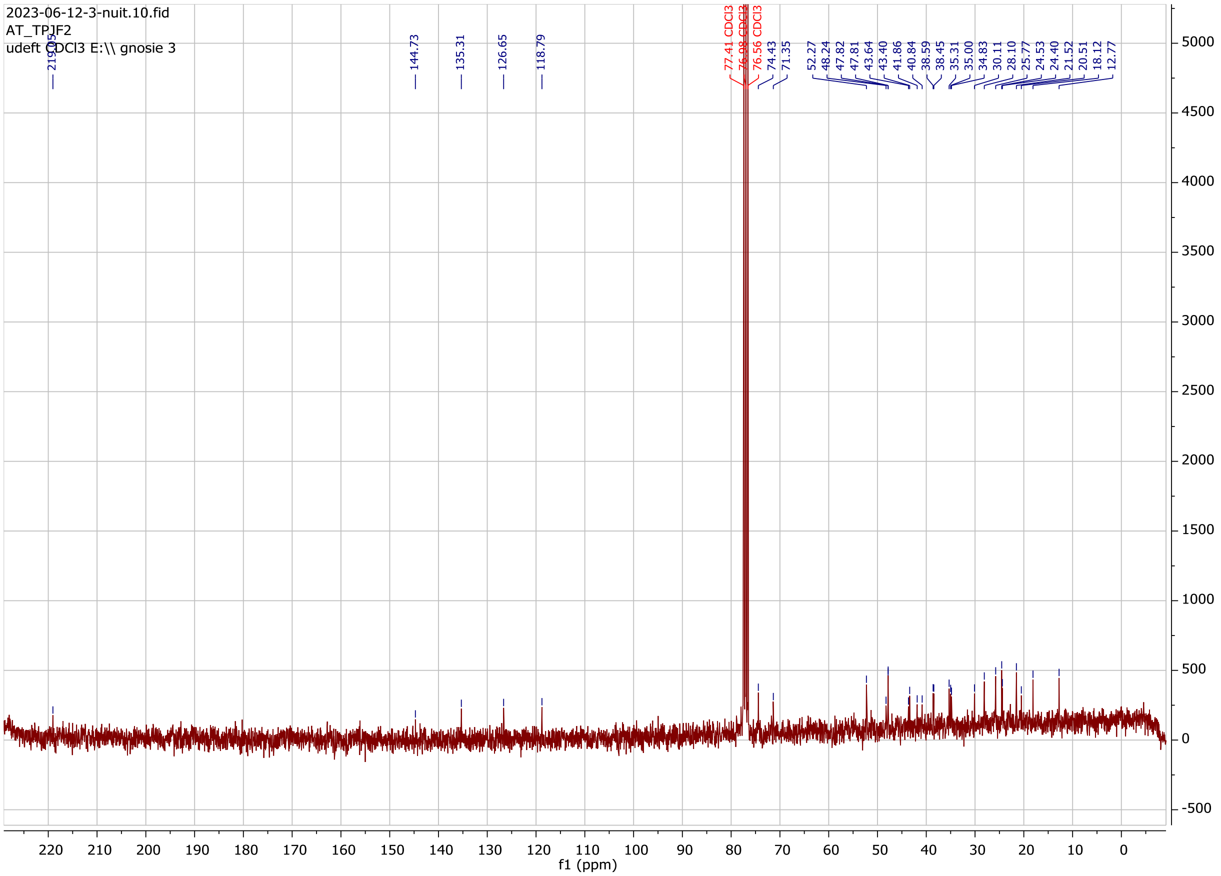

Figure S5: ^{13}C NMR spectrum of flindissone (**1**) ($CDCl_3$, 75MHz).

Biological Evaluation of Flindissone (1)

Table S2. List of Plasmids Used for Luciferase Assays

Plasmid (Vector)	Source
Full-length human LXRa (pcDNA3.1+)	Missouri S&T cDNA Resource Center (NR1H300001)
Full-length human LXRb (pcDNA3.1+)	Missouri S&T cDNA Resource Center (NR1H200000)
Human ABCA1 promoter_Luciferase (pGL4.14)	Dr. Ira G. Schulman (University of Virginia, Charlottesville, VA, USA)
hLXRa-LBD:Gal4-DBD (pCMX)	Dr. Makoto Makishima (Nihon University, Tokyo, Japan)
hLXRb-LBD:Gal4-DBD (pCMX)	Dr. Makoto Makishima (Nihon University, Tokyo, Japan)
UAS promoter_Luciferase (tk(MH1000))	from Dr. Roland Evans (Salk Institute, La Jolla, CA, USA)
pEGFP-N1	Takara Bio (Mountain View, CA, USA)

Table S3. List of Primers Used for RT-qPCR

Gene	Forward	Reverse
hABCA1	TCCCCGGTTCTGTTTTCTCC	CGCCGTGGCTGGTCATTAA
hABCG1	CACCAGCGGCCTGGAC	GTGCAAATGATGGAGC GACC
hPPIA	GCCGAGGAAAACCGTGTACT	TGTCTGCAAACAGCTCAAAGG
hSREBF1	GCTGCAGCCCCACTTCATC	TCACCAGGGTCGGCAAAG
hLDLR	CAGCTACCCCTCGAGACAGA	GCAGGCAATGCTTTGGTCTT
hMYLIP/IDOL	GACTGCCTCAACCAGGTGT	GGGAGATCCGGTTTCTCAGG
hHMGCR	AGTGAGATCTGGAGGATCCAAG	ACAAAGAGGCCATGCATTCG

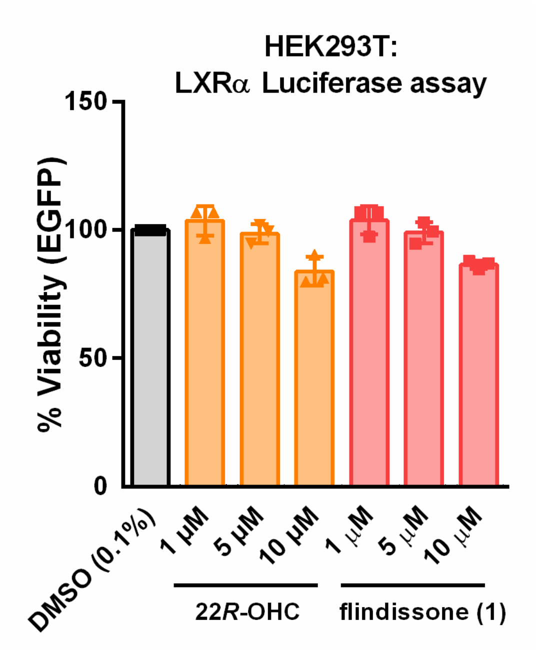

Figure S6. Cell viability relative to the DMSO control during LXRα luciferase assay as assessed by the EGFP fluorescence.

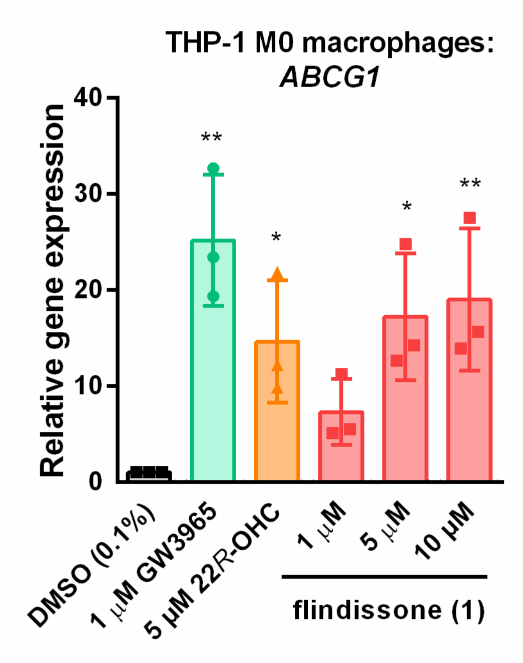

Figure S7. Flindissone-mediated ABCG1 gene regulation in THP-1 M0 macrophages. The cells were treated as described in the Experimental section and gene expression was measured by RT-qPCR. *N*=3. Mean ± SD. One-way ANOVA with Dunnett´s post hoc test. Significance indicated in comparison to the DMSO (0.1%) control: *: p = 0.05, **: p = 0.01, no indication = not significant.

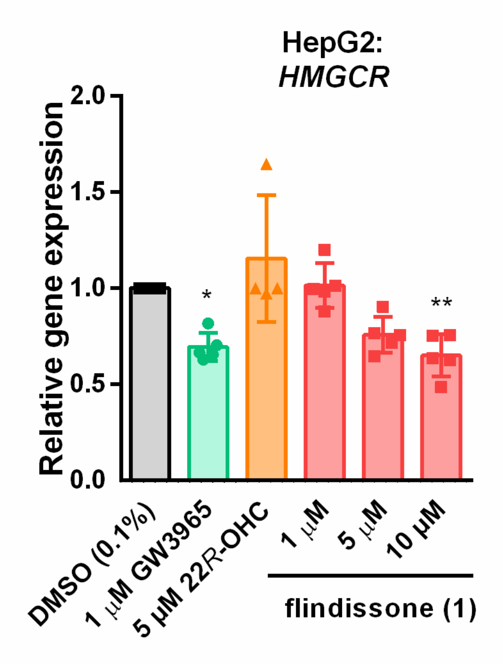

Figure S8. Flindissone-mediated HMGCR gene regulation in HepG2 cell line. The cells were treated as described in Experimental section and gene expression was measured by RT-qPCR. *N*=5. Mean ± SD. One-way ANOVA with Dunnett´s post hoc test. Significance indicated in comparison to the DMSO (0.1%) control: *: p = 0.05, **: p = 0.01, no indication = not significant.
